# Social Attention in Autism: Neural Sensitivity to Speech Over Background Noise Predicts Encoding of Social Information

**DOI:** 10.3389/fpsyt.2020.00343

**Published:** 2020-04-24

**Authors:** Leanna M. Hernandez, Shulamite A. Green, Katherine E. Lawrence, Marisa Inada, Janelle Liu, Susan Y. Bookheimer, Mirella Dapretto

**Affiliations:** ^1^ Ahmanson-Lovelace Brain Mapping Center, University of California, Los Angeles, Los Angeles, CA, United States; ^2^ Department of Psychiatry and Biobehavioral Sciences, University of California, Los Angeles, Los Angeles, CA, United States; ^3^ Staglin IMHRO Center for Cognitive Neuroscience, University of California, Los Angeles, Los Angeles, CA, United States

**Keywords:** speech, autism, voice-selective, attention, conversation, noise, aversive, sensory

## Abstract

Autism spectrum disorder (ASD) is a neurodevelopmental disorder characterized by lack of attention to social cues in the environment, including speech. Hypersensitivity to sensory stimuli, such as loud noises, is also extremely common in youth with ASD. While a link between sensory hypersensitivity and impaired social functioning has been hypothesized, very little is known about the neural mechanisms whereby exposure to distracting sensory stimuli may interfere with the ability to direct attention to socially-relevant information. Here, we used functional magnetic resonance imaging (fMRI) in youth with and without ASD (N=54, age range 8–18 years) to (1) examine brain responses during presentation of brief social interactions (i.e., two-people conversations) shrouded in ecologically-valid environmental noises, and (2) assess how brain activity during encoding might relate to later accuracy in identifying what was heard. During exposure to conversation-in-noise (*vs*. conversation or noise alone), both neurotypical youth and youth with ASD showed robust activation of canonical language networks. However, the extent to which youth with ASD activated temporal language regions, including voice-selective cortex (i.e., posterior superior temporal sulcus), predicted later discriminative accuracy in identifying what was heard. Further, relative to neurotypical youth, ASD youth showed significantly greater activity in left-hemisphere speech-processing cortex (i.e., angular gyrus) while listening to conversation-in-noise (*vs*. conversation or noise alone). Notably, in youth with ASD, increased activity in this region was associated with higher social motivation and better social cognition measures. This heightened activity in voice-selective/speech-processing regions may serve as a compensatory mechanism allowing youth with ASD to hone in on the conversations they heard in the context of non-social distracting stimuli. These findings further suggest that focusing on social and non-social stimuli simultaneously may be more challenging for youth with ASD requiring the recruitment of additional neural resources to encode socially-relevant information.

## Introduction

Autism spectrum disorder (ASD) is a common neurodevelopmental disorder characterized by difficulties in social interaction and communication, the presence of repetitive behaviors and restricted interests, as well as sensory processing atypicalities (1). Research in infants who later go on to get an ASD diagnosis has consistently shown that allocation of attention to social stimuli is disrupted early in development [for a review, see (2)]. For instance, young children with ASD fail to show a preference for listening to their mothers' voice (3), as well as to child-directed speech (4); disrupted attention to language early in life may set the stage for subsequent atypical language acquisition, as well as altered development of the neural systems responsible for language processing. Importantly, the ability to selectively attend to and learn from social interactions in one's environment often requires the simultaneous filtering out competing non-social stimuli. As heightened sensory sensitivity to mildly aversive auditory stimuli (e.g., loud noises) is observed in a significant number of children with ASD (5), we hypothesize that this may be one potential mechanism through which attention may be drawn away from social input in favor of other non-social stimuli present in the environment. Despite growing interest in the relationship between sensory processing and social impairments in ASD (6–8), little research to date has investigated how individual variability in neural responses to *simultaneous* social and non-social sensory stimuli may relate to the ability to “hone in” on socially-relevant input.

Converging neuroimaging data indicate altered brain responses to language in individuals with ASD. While ASD is characterized by a great deal of heterogeneity (9), young children with ASD who go on to have poorer language skills show hypoactivity in temporal cortex during language listening (10), as well as reduced functional connectivity between nodes of the language network (11). In children and adolescents with ASD, functional MRI (fMRI) studies have found reduced functional lateralization and increased rightward asymmetry during a variety of language processing tasks, as compared to the leftward asymmetry observed in neurotypical individuals (12–17), as well as reduced connectivity between voice-selective cortex and reward-related brain regions (18).

Importantly, however, in most real-life situations language is not heard in isolation but against the background of other competing sensory distractors (e.g., a buzzing fan, a barking dog). In neurotypical adults, the bilateral posterior superior temporal sulcus (pSTS) responds selectively to vocal stimuli, and activity in this region is reduced when voice stimuli are degraded or masked by background noise (19, 20). In contrast, individuals with ASD fail to activate voice-selective regions in the pSTS during exposure to vocal stimuli (12) and show increased recruitment of right hemisphere language homologues (21). Furthermore, the ability to detect speech-in-noise appears reduced in individuals with ASD, who are poorer at identifying speech heard in the context of background noise (22, 23). Interestingly, a recent study showed that sensory processing atypicalities modulate brain activity during language processing in youth with ASD during simultaneous processing of sarcastic remarks and distracting tactile stimulation (24). However, it has yet to be examined how sensory distractors in the *same sensory modality* as speech may affect the allocation of attention to language processing during social interactions. This type of study has implications for understanding how auditory filtering deficits may affect encoding of social information in everyday life where conversations commonly occur in the context of background noises.

In adults with ASD, heightened sensory over-responsivity (SOR)—characterized by extreme behavioral response to everyday sensory stimuli—is related to higher autism traits (25). Importantly, roughly 65% of children with ASD show atypical sensory responsivity to non-social auditory stimuli (26, 27), including a lower tolerance for loud noises (28, 29) and hypersensitivity to certain environmental noises, such as the sound of a dog barking or a vacuum cleaner (30). A growing body of neuroimaging research also suggests that children with ASD who have high levels of SOR display neural hyper-responsivity to aversive visual, tactile, and auditory stimuli in primary sensory brain regions and areas important for salience detection (31, 32), suggesting that there may be an over-allocation of attentional resources to sensory stimuli in youth with ASD. Together, these data suggest that language processing within social contexts in which there are other competing sensory stimuli—such as those that occur in the natural environment—may be particularly challenging for some individuals with ASD.

Here, we examined brain responses to auditory social and non-social stimuli in a paradigm where participants heard brief conversations between two people which were shrouded in competing environmental noises. Ecologically valid stimuli were developed to examine the effects of ASD diagnosis on neural processing of commonly encountered environmental noise, conversation, and conversation-in-noise (i.e., noise and conversation presented simultaneously). In addition, participants completed a post-scan computerized test that probed recognition of the noises and topics of conversation presented during the fMRI paradigm, thus providing a measure of attention to, and encoding of social and non-social information. We hypothesized that, relative to neurotypical youth, youth with ASD would show reduced activity in left hemisphere language cortices when listening to conversation alone, as well as increased activity in sensory cortices when exposed to aversive noise. Further, we expected that the presence of distracting noises during speech processing would result in greater activation of subcortical and cortical brain regions involved in sensory processing in youth with ASD relative to neurotypical youth. Finally, we expected that the ability to recognize details from the conversations heard in presence of background noises would be associated with increased activity in canonical left hemisphere language regions and voice-selective cortex in the pSTS in both groups, reflecting the recruitment of additional neural resources to “hone in” on social stimuli in the context of non-social distractors; to the extent that some youth with ASD may show hypersensitivity to auditory stimuli, we expect this effect would be more pronounced in this group.

## Materials and Methods

### Participants

Participants were 26 youth with ASD and 28 age-matched typically-developing (TD) youth who were recruited through referrals from the University of California, Los Angeles (UCLA) Child and Adult Neurodevelopmental (CAN) Clinic, as well as from posted advertisements throughout the greater Los Angeles area. Exclusionary criteria included any diagnosed neurological or genetic disorders, as well as structural brain abnormalities, or metal implants. ASD participants had a prior clinical diagnosis, which was confirmed using the Autism Diagnostic Observation Schedule—2nd Edition (ADOS-2) (33) and Autism Diagnostic Interview-Revised (ADI-R) (34) by licensed clinicians at the UCLA CAN Clinic. All participants had full-scale IQ above 70 as assessed by the Wechsler Abbreviated Scale of Intelligence (35) (**Table 1**). Data were originally acquired for 30 ASD and 30 TD youth, 4 ASD participants, and 2 TD participants were excluded from the final sample due to excessive head motion during fMRI data acquisition (i.e., greater than 3.5 mm of maximum relative motion; see **Table 1** for mean motion parameters in the final sample). Study procedures were approved by the UCLA Institutional Review Board and informed consent and assent to participate in this research were obtained in writing from legal guardians and study participants.

**Table 1:** Descriptive statistics.

	ASD mean (SD)	TD mean (SD)	t or *x* ^2^	
*Demographics*				
Sex (N male)	19	17	0.93	
Age	13.75 (2.98)	13.78 (2.66)	−0.04	
Full IQ	102.42 (14.92)	113.11 (13.05)	−2.79	**
Nonverbal IQ	107.96 (17.61)	112.61 (12.69)	−1.11	
Verbal IQ	97.42 (14.30)	110.64 (13.42)	−3.50	***
SRS Total T-Score	68.77 (12.06)	44.46 (5.90)	9.30	***
SRS Social Awareness T-Score	67.50 (11.19)	45.18 (6.98)	8.72	***
SRS Social Cognition T-Score	67.27 (12.54)	44.54 (7.30)	8.06	***
SRS Social Communication T-Score	67.58 (12.75)	44.57 (5.76)	8.44	***
SRS Social Motivation T-Score	61.77 (11.80)	47.01 (7.41)	5.44	***
*Motion*				
Mean absolute motion (mm)	0.44 (0.28)	0.42 (0.28)	0.36	
Max absolute motion (mm)	1.76 (1.65)	1.39 (1.23)	0.92	
Mean relative motion (mm)	0.14 (0.07)	0.14 (0.06)	−0.02	
Max relative motion (mm)	1.21 (1.08)	0.92 (0.78)	1.11	
*Post-scan test: percent correct*				
Conversations, alone condition				
Easy questions	75.76% (20.77)	81.95% (17.35)	−1.18	
Hard questions	79.58% (16.92)	76.02% (22.82)	0.65	
Conversations, conversation-in-noise condition				
Easy questions	66.43% (16.36)	69.59% (16.36)	−0.60	
Hard questions	61.73% (25.46)	73.16% (25.46)	−1.60	
				
*Post-Scan Test: discriminative accuracy (d')*				
Conversations, alone condition	1.73 (0.97)	1.92 (1.01)	−0.71	
Conversations, conversation-in-noise condition	1.28 (0.86)	1.59 (0.99)	−1.21	

**p < 0.01, ***p < 0.001 ASD, autism spectrum disorder; TD, typically developing; IQ, intelligence quotient.

### Behavioral Measures

Social functioning was assessed in both ASD and TD youth using the Social Responsiveness Scale—2^nd^ Edition (SRS-2) (36). The SRS-2 is intended for use in both neurotypical populations and individuals with ASD and provides a measure of the severity of social impairment associated with autism. In the current study, we examined the relationship between t-scores for the socially-relevant subscales of the SRS-2 (i.e., social awareness, social cognition, social communication, and social motivation) and neural activity during conversation-in-noise listening.

### Experimental Design

**Figure 1:**
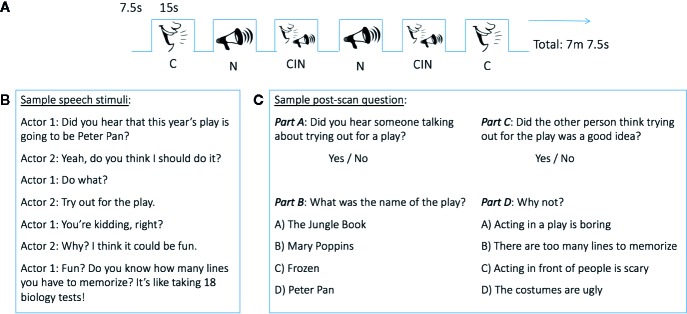
Experimental design. **(A)** Block design functional MRI (fMRI) task. **(B)** Example of a conversation heard during fMRI data acquisition. **(C)** Sample of post-scan questions. CIN, conversation-in-noise; C, conversation; N, noise.

During the fMRI scan, auditory stimuli were presented according to a canonical block design (**Figure 1A**) using E-Prime 2.0 Software on a Dell Latitude E6430 laptop computer. Each block consisted of 15 s of auditory stimulus presentation alternating with 7.5 s of rest. A crosshair was presented at the center of a white screen throughout the duration of the scan. Blocks consisted of three types: conversation (C), noise (N), and conversation-in-noise (CIN; i.e., conversation and noise presented simultaneously). Stimuli were ecologically valid and mimicked those encountered in everyday life, whereby one overhears two people engaged in a conversation that is shrouded by competing auditory stimuli, thus forcing the listener to “hone in” on the socially relevant speech. Inspiration for conversation topics were taken from scripted television series focusing on childhood/adolescence (**Figure 1B**). Speech passages were recorded by two actors (one male, one female) using GarageBand 6.0.5 and an Apogee MiC digital microphone connected to a Macintosh computer. Noise stimuli were downloaded from Freesound.org. Selection of noise stimuli ensured that they were ecologically valid (i.e., commonly encountered in everyday life). The aversive nature of the selected noises was rated in an independent sample (N=30) using a 7-point Likert scale (1=not aversive, 7=extremely aversive); the final 12 noise stimuli used in the fMRI paradigm were rated as moderately aversive (rating M=4.7, range 3.6–5.5) and included such sounds as a jackhammer, a police siren, and a blender. Root-mean-square amplitude was normalized across all stimuli to control for loudness. Stimuli were counterbalanced such that half of the participants heard a given conversation without noise, whereas the other half of participants heard the same conversation masked by noise (i.e., in the CIN condition). Likewise, for any given noise, half of participants heard the noise alone, while the other half heard the noise in the CIN condition. Each block type (C, N, CIN) was presented six times; order was counterbalanced across subjects. The total run time was 7 min and 7.5 s. Prior to the fMRI scan, participants were told that they would hear some people talking and some noises; they were instructed to just listen and look at the crosshair on the screen. Participants were not specifically instructed to pay attention to what was said, as we wanted the paradigm to have high ecological validity by mimicking situations encountered in everyday life when we may overhear others talking and are not explicitly asked to pay attention or remember what was said.

To assess the participants' ability to recognize stimuli presented in the three experimental conditions, and thus gain a proximal measure of in-scanner attention, a brief post-MRI scanning questionnaire was administered using E-Prime 2.0 Software on a Dell Latitude E6430 laptop computer. During this post-scanning test, participants heard and read questions about the conversations and noises they were exposed to during the fMRI data acquisition, interspersed with foils (i.e., with questions about conversations and noises they did not hear). For each conversation and noise stimulus presented during the fMRI scan, participants were first asked to answer a question about whether they heard such a particular conversation topic or noise. For the conversations, the post-scan test was tiered such that if a participant's yes/no response to this initial question was correct (**Figure 1C**, top), a more nuanced question about that conversation was then presented (**Figure 1C**, bottom). Incorrect responses to the initial yes/no questions resulted in being presented the next set of questions about a different conversation topic. Participant responses were recorded in E-Prime. A sensitivity index (d') was calculated to assess the ability of youth to discriminate between topics of conversation heard during MRI scanning and foils. d' was calculated as the standardized (i.e., z-transformed) proportion of hits minus the standardized proportion of false alarms.

### MRI Data Acquisition

MRI data were collected on a 3.0 Tesla Siemens Prisma MRI Scanner using a 64-channel head coil. For each subject, a multi-slice echo-planar (EPI) sequence was used to acquire functional data: 595 volumes; repetition time (TR) = 720 ms; multiband acceleration factor = 8; matrix size = 104 x 104; field of view (FOV) = 208 × 208 mm; in-plane resolution = 2 × 2 mm; slice thickness = 2 mm, no gap; 72 slices; bandwidth = 2,290 Hz per pixel; echo time (TE) = 37 ms. Visual and auditory stimuli were presented *via* magnetic resonance compatible goggles and headphones (Optoacoustics LTD, Or Yehuda, Israel). Subjects wore earplugs and headphones to lessen scanner noise.

### Functional MRI Data Analysis

Data were processed using FSL (FMRIB's Software Library, www.fmrib.ox.ac.uk/fsl) (37) and AFNI (Analysis of Functional NeuroImages) (38). Functional data were motion corrected to the average functional volume with FSL's Motion Correction Linear Registration Tool (MCFLIRT) (39) using sinc interpolation and skull stripped using FSL's Brain Extraction Tool (BET) (40). Time series statistical analyses were run in FSL's FMRI Expert Analysis Tool (FEAT) version 6.0. Functional images were spatially smoothed [full width at half maximum (FWHM) 5 mm] and a temporal high pass filter of 67.5 s was applied. Functional data were linearly registered to the Montreal Neurological Institute (MNI) 2 mm standard brain with 12° of freedom. Motion outliers were identified using FSL's motion outliers tool (comparing the root mean square intensity difference from the center volume to identify outliers) and were included as a confound explanatory variable in the single subject analyses; there was no difference in the mean number of volumes censored between ASD and TD participants (p=0.31). Condition effects were estimated by convolving a box-function for each condition with a double-gamma hemodynamic response function, along with the temporal derivative. Each condition was modeled with respect to resting baseline (C, N, CIN); single-subject models were combined into a group-level mixed effects model (FLAME1+2). Verbal IQ was entered as a covariate in all group-level analyses. Within-group and between-group maps were pre-threshold masked by grey matter and thresholded at z > 3.1 (p < 0.001), cluster-corrected for multiple comparisons at p < 0.05. Between-group comparisons (i.e., ASD *vs*. TD) were masked by the sum of within-group activity for each condition of interest.

### Statistical Analysis

Two-tailed t-tests were performed to assess between-group differences in age, IQ, and motion parameters. To test whether participant's discriminative accuracy (d') for identifying the topics of conversation varied as a function of diagnostic group, condition, or question, a repeated measures ANOVA was conducted with group (i.e., ASD *vs*. TD) as the between-subjects factor and condition (i.e., N, C, CIN) as within-subjects factors. To further examine differences in behavioral performance, we also ran separate repeated measures ANOVAs comparing percent of correct responses for easy (yes/no) and hard (multiple-choice) questions separately with group (i.e., ASD *vs*. TD) as the between-subjects factor and condition (i.e., C *vs*. CIN) as the within-subjects factor.

## Results

### Demographics

There were no statistically significant differences between ASD and TD youth in sex, age, and non-verbal IQ, or across any of the four motion parameters tested (**Table 1**). Two-sample t-tests revealed significant differences in full-scale and verbal IQ between ASD and TD youth, whereby TD youth had higher IQ relative to their ASD counterparts. As expected, ASD and TD youth also had significantly different t-scores on the social awareness, social cognition, social communication, and social motivation subscales of the Social Responsiveness Scale (SRS), as well as differences in SRS Total t-scores, indicative of poorer parent-reported social functioning in youth with ASD.

### Post-Scan Recognition Test

To assess participants' ability to discriminate between what was actually heard *vs*. foils (i.e., correctly identifying a conversation, or noise, that was heard—“hits”—*vs*. incorrectly endorsing a conversation or noise that was not heard—“false alarms”), we calculated a sensitivity index (d') for each participant. In ASD youth, mean d' was 0.64, 0.59, 1.73, 1.28, for noises heard in the alone condition, noises heard in the conversation-in-noise condition, conversations head in the alone condition, and conversations heard in the conversation-in-noise condition, respectively. Likewise, mean d' in TD youth was 0.65, 0.67, 1.92, and 1.59 for noises heard in the alone condition, noises heard in the conversation-in-noise condition, conversations head in the alone condition, and conversations heard in the conversation-in-noise condition, respectively. A repeated-measures ANOVA was performed to test the interaction between group x condition. This analysis revealed no significant group x condition interaction [F(3,156)=0.56, p=0.64)] or main effect of Group [F(1,52)=0.83, p=0.37)]. However, the main effect of condition was significant [F(3,156)=46.46, p < 0.001)]; pairwise comparisons showed that both ASD and TD participants had higher accuracy (d') for conversations heard in the alone condition as compared to noises heard in the alone condition, as well as higher accuracy for conversations than noises when these were heard in the conversation-in-noise condition.

In order to further examine differences in behavioral performance, we also compared subjects' percent accuracy using separate repeated measures ANOVAs for easy (yes/no) and hard (multiple-choice) questions. For the easy questions, the main effect of condition was significant [F(1,52)=19.77, p < 0.001], whereby both groups were more accurate at identifying topics of conversation heard in the conversation alone condition than in the conversation-in-noise condition. However, there was no significant group x condition interaction [F(1,52)=0.38, p=0.54] or main effect of group [F(1,52)=1.02, p=0.32)]. For the hard (multiple-choice) questions, there was also a main effect of condition [F(1,52)=10.00, p < 0.01)], whereby both groups were more accurate at identifying topics of conversation heard in the conversation alone condition. However, while there was no main effect of group [F(1,52)=0.51, p=0.48)], there was a significant group x condition interaction [F(1,52)=5.53, p=0.02)]. *Post hoc* tests showed that while the ASD and TD groups did not differ in percent accuracy for the conversation alone or conversation-in-noise conditions, the ASD group was significantly more accurate for the conversation alone condition than for the conversation-in-noise (p < 0.01); this was not the case for TD youth (p > 0.05).

### Functional MRI Results

#### Within-Condition Analyses

**Figure 2:**
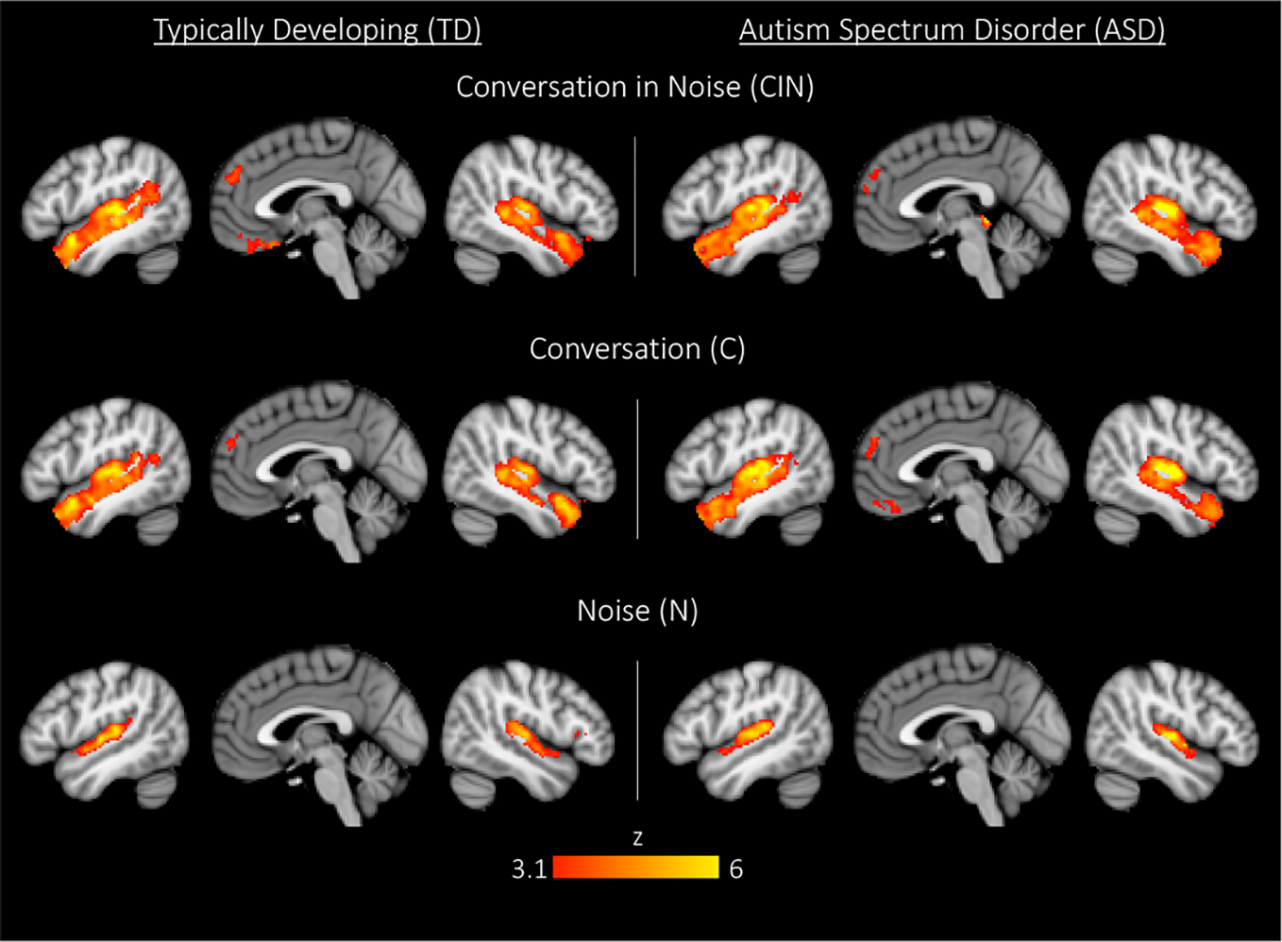
Whole-brain activation in typically developing (TD) youth and youth with autism spectrum disorder (ASD) during exposure to conversation-in-noise (CIN), conversation (C), and noise (N). Maps are thresholded at z > 3.1, corrected for multiple comparisons at the cluster level (p < 0.05).

Across each of the three conditions, both youth with ASD and TD youth showed the expected activity in bilateral Heschl's gyrus, superior temporal gyrus, planum temporal, and planum polare (**Figure 2**, **Table 2**). During exposure to conversation-in-noise (CIN) and conversation alone (C), both groups showed robust activation in auditory and language cortices, including bilateral superior temporal gyrus (STG), middle temporal gyrus, temporal pole, left angular gyrus, and superior frontal gyrus. Activity in ventromedial prefrontal cortex, a region involved in theory of mind and mentalizing, was observed in TD youth in the CIN condition, and in ASD youth in the C condition. In contrast to the extended network of regions activated during conditions in which speech was presented (i.e., CIN and C), brain activity during the noise condition (N) was restricted to primary and secondary auditory cortices; ASD youth showed additional activation in right inferior frontal gyrus and pars triangularis. No between-group differences were observed for any of the three experimental conditions at this statistical threshold (z > 3.1, p < 0.05).

**Table 2:** Montreal Neurological Institute (MNI) coordinates for each condition (conversation-in-noise, CIN; conversation, C; noise, N) compared to baseline.

		*Conversation-in-noise (CIN)*										*Conversation (C)*										*Noise (N)*								
		ASD					TD					ASD					TD					ASD					TD			
		Max z	MNI peak (mm)				Max z	MNI peak (mm)				Max z	MNI peak (mm)				Max z	MNI peak (mm)				Max z	MNI peak (mm)				Max z	MNI peak (mm)		
			X	Y	Z			X	Y	Z			X	Y	Z			X	Y	Z			X	Y	Z			X	Y	Z
Angular gyrus	L	3.57	−60	−58	22		5.27	−58	−56	20		3.66	−60	−58	22		3.83	−58	−58	16										
Central opercular cortex	L	4.39	−52	−12	10		4.12	−58	−10	8		4.79	−60	−20	14		4.21	−58	−10	8		3.85	−60	−16	12		3.59	−50	−20	14
Central opercular cortex	R	4.63	50	−12	10		4.24	56	−6	6		4.81	48	−12	10		4.00	54	−10	8		3.58	50	−10	8		3.55	56	−6	6
Frontal operculum cortex																											3.51	40	24	2
Frontal orbital cortex	R						4.48	40	30	−18							3.26	40	26	−20										
Frontal pole	L	3.54	−2	60	20		5.06	−12	50	34		3.88	−10	58	28		4.83	−12	52	32										
Frontal pole	R	3.77	4	60	20												4.01	12	50	36										
Frontal medial cortex	L						3.48	−2	36	−24		4.07	−4	38	−20															
Frontal medial cortex	R						4.41	2	44	−16		4.10	4	38	−20															
Heschl's gyrus	L	5.78	−50	−22	8		6.79	−40	−24	10		6.73	−38	−26	12		7.15	−40	−24	10		6.13	−44	−18	4		6.03	−44	−18	4
Heschl's gyrus	R	6.43	44	−16	6		5.75	50	−20	8		6.71	48	−14	6		5.62	42	−22	10		6.21	46	−14	6		5.49	44	−18	8
Insular cortex	L						3.74	−40	−16	6		3.68	−42	−12	4		3.69	−40	−16	6		3.88	−40	−4	−12		3.82	−42	−6	−6
Insular cortex	R											3.20	42	−12	6							3.17	40	−6	−10					
Lateral occipital cortex	L	3.11	−58	−64	24																									
Middle temporal gyrus	L	5.01	−58	−2	−16		5.58	−52	−28	−6		5.55	−56	−2	−18		5.59	−66	−16	−16										
Middle temporal gyrus	R	5.32	50	−24	−6		5.26	58	−32	−2		4.98	50	−24	−6		5.16	64	−12	−10										
Paracingulate gyrus	L						3.79	−4	48	26																				
Paracingulate gyrus	R	3.78	4	52	20												3.30	4	50	22										
Parietal operculum cortex	L	5.22	−48	−28	14		3.54	−46	−30	14		5.59	−48	−28	14		3.70	−58	−30	16		5.59	−46	−30	14		3.93	−48	−28	14
Parietal operculum cortex	R	4.31	44	−24	16		3.77	46	−26	16		3.87	44	−24	16		3.84	48	−26	16							4.12	54	−24	16
Planum polare	L	4.82	−44	−18	−4		4.08	−48	0	−12		4.65	−42	2	−20		4.06	−48	0	−12		4.47	−46	0	−12		5.40	−46	−8	−6
Planum polare	R	4.25	46	−12	−4		3.97	48	2	−14		4.10	58	2	0		4.26	46	4	−16		4.19	44	4	−16		4.47	46	−4	−10
Planum temporale	L	6.55	−56	−28	8		6.25	−52	−26	6		7.23	−56	−28	8		6.01	−62	−20	8		6.27	−44	−32	10		5.74	−52	−26	8
Planum temporale	R	6.18	58	−24	10		6.02	62	−20	8		6.00	44	−30	12		6.00	62	−20	8		4.65	60	−22	10		6.64	58	−26	12
Postcentral gyrus	L	3.25	−64	−16	16							3.63	−64	−16	16															
Postcentral gyrus	R	3.10	66	−14	16							3.13	66	−14	16															
Subcallosal cortex	L						4.39	−2	20	−24																				
Subcallosal cortex	R						4.71	2	20	−24																				
Superior frontal gyrus	L	3.43	−2	52	34		4.59	−4	52	28		5.54	−4	50	32		4.70	−6	52	30										
Superior frontal gyrus	R	5.77	4	52	30							3.35	2	48	36		3.66	6	50	36										
Superior temporal gyrus	L	6.65	−66	−28	10		6.23	−66	−24	0		6.39	−66	−28	10		6.13	−66	−18	4		4.10	−66	−26	10		4.89	−66	−26	10
Superior temporal gyrus	R	6.25	68	−18	4		6.72	66	−20	2		6.71	68	−18	4		6.96	66	−20	2		5.18	68	−20	2		7.17	70	−24	2
Supramarginal gyrus	L	4.38	−58	−46	22		3.99	−64	−46	16		3.93	−64	−46	16		3.17	−64	−48	16										
Supramarginal gyrus	R	3.63	66	−40	10		3.65	66	−40	10																				
Temporal pole	L	5.69	−44	16	−20		6.10	−46	10	−20		5.90	−58	6	−16		5.88	−50	10	−20		4.50	−52	6	−8		3.81	−52	6	−6
Temporal pole	R	7.08	42	14	−22		5.51	56	10	−20		5.38	52	12	−26		6.63	52	14	−18		3.60	58	8	−8		3.52	56	8	−6
Thalamus	L	3.63	−10	−32	0																									
Thalamus	R	3.70	10	−32	0																									

Region labels refer to Harvard Oxford Atlas, thresholded at 50%.

#### Between-Condition Analyses

**Figure 3:**
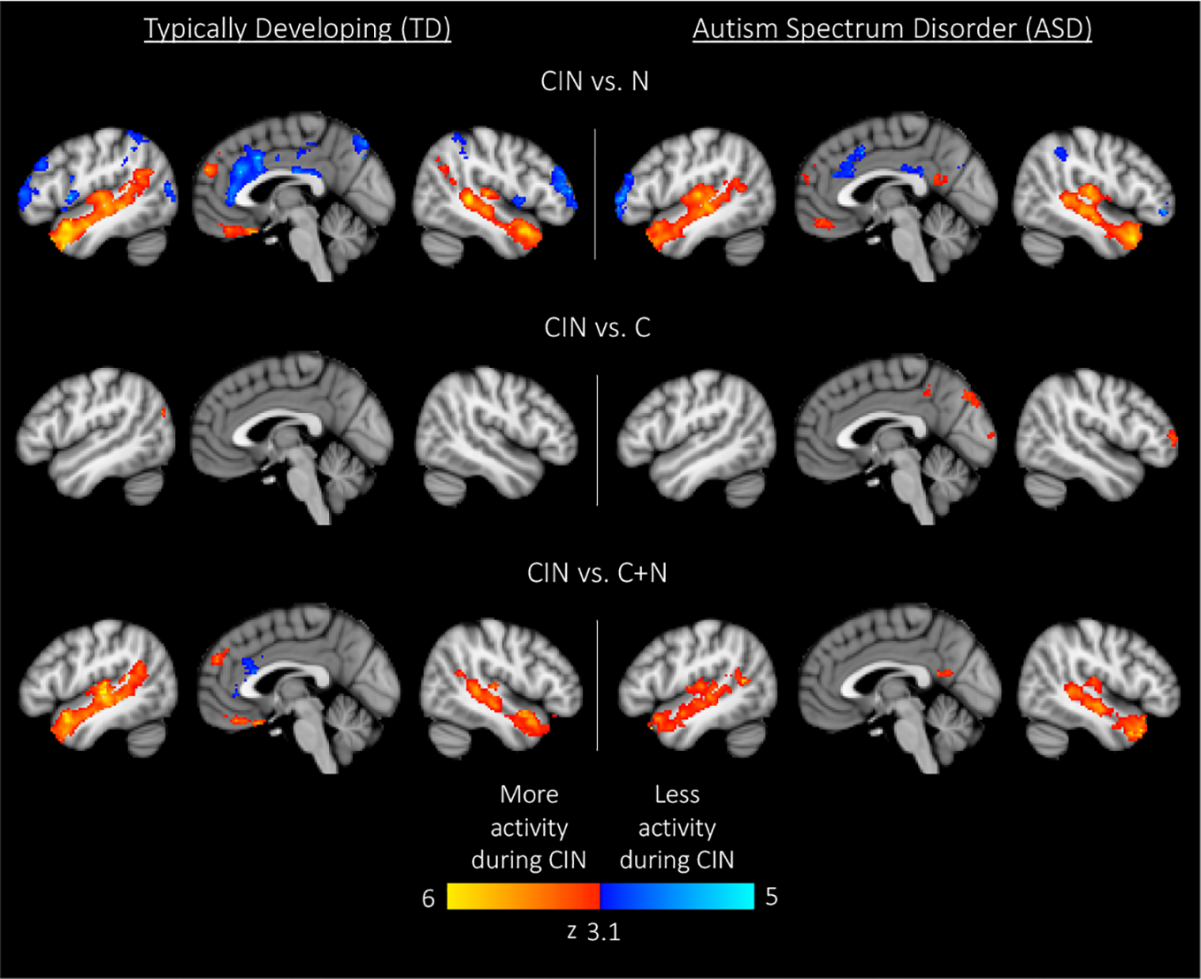
Within-group results for comparisons between experimental conditions. Maps are thresholded at z > 3.1, corrected for multiple comparisons at the cluster level (p < 0.05). CIN, conversation-in-noise; N, noise; C, conversation.

Here we compared brain activity between experimental conditions. First, we examined differences in brain activity when listening to conversation-in-noise relative to listening to noise alone (CIN > N). For this contrast, both TD and ASD youth showed increased activity in bilateral temporal pole, superior temporal gyrus, Heschl's gyrus, superior frontal gyrus, and medial prefrontal cortex (**Figure 3**, **Table 3**), consistent with increased attention to language stimuli in the CIN condition. TD youth also showed activation in the right angular gyrus and bilateral hippocampus, whereas ASD youth showed significant activation in the precuneus. No regions showed significant between-group differences when comparing CIN and N conditions.

**Table 3:** Montreal Neurological Institute (MNI) coordinates for between-condition contrasts.

		*CIN > N*										*CIN > C*										*CIN > C+N*								
		ASD					TD					ASD					TD					ASD					TD			
		Max z	MNI peak (mm)				Max z	MNI peak (mm)				Max z	MNI peak (mm)				Max z	MNI peak (mm)				Max z	MNI peak (mm)				Max z	MNI peak (mm)		
			X	Y	Z			X	Y	Z			X	Y	Z			X	Y	Z			X	Y	Z			X	Y	Z
Amygdala	L						4.78	−28	−6	−20																				
Amygdala	R						4.74	24	−6	−20																				
Angular gyrus	L	3.89	−62	0	20		4.64	−58	−56	20												3.32	−62	−54	20		5.92	−56	−54	20
Angular gyrus	R						5.39	52	−54	20												4.35	62	−48	22		4.15	54	−50	18
Central opercular cortex	L	3.95	−52	−12	10		4.52	−58	−10	8												3.64	−50	−8	6		3.96	−52	−10	8
Central opercular cortex	R	4.33	48	−16	12		3.19	62	−8	8												3.57	48	−16	12		4.25	62	−8	8
Cingulate gyrus posterior	L	3.83	−2	−50	22																	4.26	−2	−50	18					
Cingulate gyrus posterior	R	3.68	2	−50	20																	3.24	2	−48	18					
Cuneal cortex	L											3.77	−2	−82	34															
Frontal medial cortex	L	4.46	−4	42	−16		4.45	−2	38	−22																	4.6	−2	36	−22
Frontal medial cortex	R	4.93	2	44	−16		6.46	2	42	−22																	5.05	4	40	−22
Frontal orbital cortex	L	3.65	−38	20	−20																									
Frontal orbital cortex	R						3.14	40	26	−20																	3.19	44	28	−18
Frontal pole	L	3.57	−4	64	24		3.7	−10	58	28																				
Frontal pole	R	4.11	12	42	48							4.84	38	44	6							4.03	12	48	46					
Fusiform cortex	L						3.69	−40	−18	−24																				
Heschl's gyrus	L	5.11	−48	−18	8		5.09	−40	−22	8												4.45	−50	−22	8		6.35	−46	−24	10
Heschl's gyrus	R	5.25	48	−20	10		5.67	50	−20	8												4.04	48	−20	10		4.66	50	−20	8
Hippocampus	L						4.85	−26	−8	−22																				
Hippocampus	R						4.66	26	−8	−20																				
Inferior temporal gyrus	L						3.61	−56	−18	−28																	3.58	−56	−20	−26
Lateral occipital cortex	L	3.86	−56	−64	24		4.2	−50	−62	26		3.7	−12	−82	46		4.41	−48	−74	26		3.57	−56	−62	26					
Lateral occipital cortex	R																					4.79	54	−64	18					
Middle temporal gyrus	R	5.26	50	−24	−6		6.31	−54	−26	−8												4.02	−56	−32	−4		5.84	−54	−28	−6
Middle temporal gyrus	L	5.55	−56	0	−28		4.99	64	−12	−10												5.16	52	−20	−8		4.9	62	−30	−4
Occipital pole	L											4.74	−12	−96	−2															
Occipital pole	R											3.51	2	−96	6															
Paracingulate gyrus	L	3.26	−6	50	20		3.32	−2	48	26																	3.31	−2	48	26
Paracingulate gyrus	R	3.18	4	52	20																	3.27	4	42	34					
Parahippocampal gyrus	L						4.16	−20	−26	−18																				
Parietal operculum Cortex	L	4.11	−42	−34	16																	3.18	−42	−34	16					
Parietal operculum Cortex	R	3.45	48	−22	16		3.75	44	−24	16																				
Planum polare	L	3.43	−54	−2	0		3.99	−44	−2	−18												3.18	−48	−4	−8		4.04	−44	0	−18
Planum polare	R	3.56	58	2	0		4.07	46	4	−16												3.62	46	0	−16		4.54	48	2	−14
Planum temporale	L	5.61	−54	−28	8		5.92	−62	−20	8												5.24	−54	−28	8		6.15	−62	−20	8
Planum temporale	R	4.39	62	−20	8		4.39	62	−18	8												3.86	62	−20	8		4.19	62	−18	8
Precuneus cortex	L	3.59	−2	−60	22							4.05	−12	−64	22							3.75	−2	−58	18					
Precuneus cortex	R	4.07	4	−56	22							4.06	8	−54	50							4.07	2	−58	18					
Subcallosal cortex	L						5.07	−2	20	−24																	4.84	−2	20	−24
Subcallosal cortex	R						5.61	2	24	−26																	4.66	2	20	−24
Superior frontal gyrus	L	3.12	−4	54	24		5.57	−4	54	24																	4.69	−4	42	38
Superior frontal gyrus	R	4.79	2	50	36																	4.03	2	42	40		3.29	4	50	32
Superior temporal gyrus	L	5.7	−62	−26	0		6.59	−64	−18	−4												5.47	−64	−26	2		6.01	−64	−18	−4
Superior temporal gyrus	R	5.31	54	−18	−6		6.34	58	−18	−4												5.26	52	−12	−10		6.06	58	−20	−2
Supramarginal gyrus	L	4.33	−64	−46	16		3.22	−64	−48	16												4.08	−58	−46	22		4.25	−64	−46	16
Supramarginal gyrus	R																					3.46	66	−38	18		3.37	66	−40	10
Temporal pole	L	5.19	−32	14	−28		6.47	−50	12	−36												5.74	−46	16	−20		6.52	−50	8	−22
Temporal pole	R	5.84	48	18	−30		5.63	50	14	−22												7.31	50	18	−30		6.04	58	8	−18

Region labels refer to Harvard Oxford Atlas, thresholded at 50%.

Next, we assessed differences in brain activity when listening to conversation-in-noise *versus* conversation alone (CIN > C). For this contrast, TD youth showed increased activity in lateral occipital cortex, whereas ASD youth had increased activity in right frontal pole, precuneus, and occipital pole (**Figure 3**, **Table 3**). Between-group comparisons revealed that the ASD group had greater activity in primary visual cortex and precuneus relative to TD youth for the contrast of CIN > C; there were no brain regions where TD youth showed greater activity relative to ASD youth (**Table 4**). No brain regions showed greater activity when listening to conversation alone *vs*. conversation-in-noise (i.e., C > CIN).

**Table 4:** Montreal Neurological Institute (MNI) coordinates for between-condition between-group contrasts.

		*CIN > C*			
		ASD > TD			
		Max z	MNI peak (mm)		
			X	Y	Z
Cuneal cortex	L	3.47	−2	−86	34
Occipital pole	L	3.6	−10	−94	0
Precuneus cortex	L	3.51	−6	−64	30

Region labels refer to Harvard Oxford Atlas, thresholded at 50%.

Lastly, to tap into the neural correlates of social attention (i.e., selective attention to speech in the context of background noise), we examined brain activity specifically associated with listening to conversation-in-noise, above and beyond activity observed for the conversation and noise alone conditions (CIN > C+N). For this contrast, both TD and ASD youth displayed activity in brain regions involved in auditory and language processing as well as theory of mind (i.e., angular gyri, superior frontal gyrus, and superior temporal regions); ASD youth displayed additional activity in the precuneus whereas TD youth showed activity in ventral medial frontal cortex (**Figure 3**, **Table 3**). No significant between-group differences were observed for this contrast.

#### Brain Activity Predicting Post-Scan Performance

**Figure 4:**
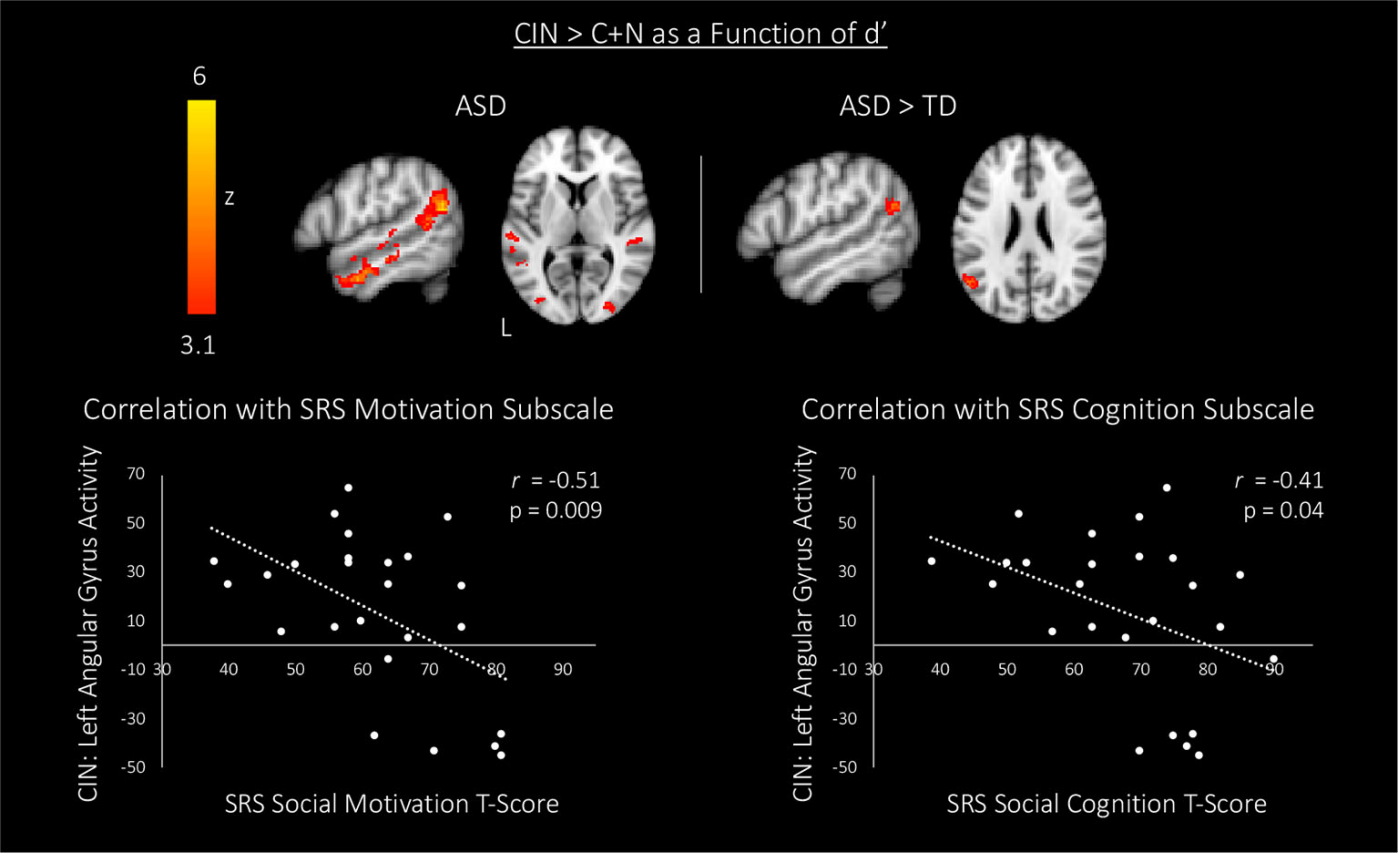
Top: associations between brain activity (CIN > C+N) and discriminative accuracy (i.e., d') for topics of conversation heard in the CIN condition. Maps are thresholded at z > 3.1, corrected for multiple comparisons at the cluster level (p < 0.05). Bottom: correlations between blood oxygen level dependent (BOLD) signal response for the CIN condition and scores on two subscales of the SRS in autism spectrum disorder (ASD) youth. CIN, conversation-in-noise; N, noise; C, conversation; SRS, Social Responsiveness Scale.

In an attempt to identify the neural substrates of social attention, we assessed how brain activity during the fMRI scan might predict accuracy in the post-scan test by entering d' as a regressor of interest in bottom-up regression analyses. We focused these analyses on our primary contrast of interest—CIN > C+N—in order to examine how d' related to brain activity specifically associated with processing conversation-in-noise *above and beyond* brain activity associated with processing conversation and noise alone. Whereas TD youth with higher d' showed selective activation of left posterior superior temporal sulcus (pSTS; i.e., voice-selective cortex), ASD youth with higher d' showed widespread increased activity primarily in language areas (**Figure 4**, **Table 5**). Direct between-group comparisons showed that, relative to TD youth, ASD youth with higher d' showed significantly greater activity in speech-processing cortex in the left angular gyrus; there were no significant results for the reverse contract. To interpret the ASD > TD effect, we examined how activity in this speech-processing region while listening to conversation-in-noise might be related to social functioning in ASD youth. Parameter estimates of activity during the CIN condition were extracted from this region and correlated with scores from the SRS subscales. Higher activity in this left speech-processing region in ASD youth was associated with lower scores on the social motivation (*r*=−0.51, p=0.009) and social cognition (*r*=−0.41, p=0.04) SRS subscales, indicating more typical patterns of behavior.

**Table 5:** Montreal Neurological Institute (MNI) coordinates for brain activity associated with discriminative accuracy (d') for topics of conversation heard in the conversation-in-noise (CIN) condition.

CIN > C+N																		
		ASD + TD				ASD				TD				ASD > TD			
		Max z	MNI peak (mm)			Max z	MNI peak (mm)			Max z	MNI peak (mm)			Max z	MNI peak (mm)		
			X	Y	Z		X	Y	Z		X	Y	Z		X	Y	Z
Angular gyrus	L	4.98	−52	−60	20	5.37	−56	−58	26					4.07	−50	−60	24
Angular gyrus	R					3.82	46	−50	28								
Frontal orbital cortex	R	4.44	44	24	−14	4.26	44	24	−14								
Fusiform cortex	L													3.86	−34	−58	−16
Lateral occipital cortex	L	3.16	−54	−64	26	5.48	−54	−64	20					4.74	−48	−64	26
Lateral occipital cortex	R					4.09	42	−84	−2								
Middle temporal gyrus	L	4.95	−62	−10	−8	5.15	−54	−4	−28	3.74	−52	−34	−4				
Middle temporal gyrus	R	4.79	56	2	−20	5.02	56	2	−30								
Occipital pole	L					4.07	−32	−94	−10								
Occipital pole	R					4.47	36	−92	−2								
Parietal operculum cortex	R					4.19	44	−32	20								
Planum temporale	L	4.00	−60	−26	6	4.37	−62	−24	10								
Planum temporale	R	4.34	52	−30	16	4.82	60	−30	16								
Precentral gyrus	R					4.17	54	−2	42								
Superior temporal gyrus	L	5.47	−62	−26	2	3.62	−58	−38	6	3.13	−60	−44	8				
Superior temporal gyrus	R	4.52	46	−32	4	3.55	52	−32	4								
Supramarginal gyrus	L	5.31	−56	−46	10	3.89	−60	−46	22								
Temporal pole	L	4.71	−44	4	−20	4.43	−44	14	−38								
Temporal pole	R	5.17	58	8	−20	4.7	56	10	−30								

Region labels refer to Harvard Oxford Atlas, thresholded at 50%.

## Discussion

Here, we examined neural activity in response to *ecologically valid* social and non-social stimuli in youth with and without ASD to elucidate the neural mechanisms through which attention may be drawn away from socially-relevant information in the presence of distracting sensory stimulation in individuals with ASD. To do so, we employed a novel paradigm whereby participants heard naturalistic conversations in the context of common environmental noises that are often in the background of everyday social interactions. Overall, both youth with ASD and typically-developing youth showed a similar pattern of brain activity in auditory and language networks when listening to conversations presented alone and conversations presented with background noise; further, minimal differences were observed between diagnostic groups when comparing brain activity during listening to conversations alone *versus* conversations shrouded in noise. When we honed in on neural mechanisms underlying the ability to later recognize the topics of conversations that were heard in the presence of background noise, we found that higher recognition accuracy was associated with greater activity in left hemisphere voice-selective cortex in typically-developing youth. In contrast, in youth with ASD, better recognition accuracy was associated with increased activity in a larger network of regions subserving language processing, with significantly greater activity observed in left speech-processing cortex relative to typically-developing youth. Furthermore, we found that increased activity in this left-hemisphere speech-processing region when listening to conversations masked in noise was related to better social motivation and social cognition in ASD youth.

At the behavioral level, youth with and without ASD were equally accurate at discriminating noises *vs*. foils (d'), regardless of whether these were presented alone or simultaneously with conversations. As expected, accuracy in discriminating what was heard during the conversations (*vs*. foils) was overall higher in typically-developing youth, compared to youth with ASD, both when the conversations were presented alone or in the context of background noise; however, these differences were not statistically significant. Notably, we deliberately did not alert participants to pay attention to what was heard in the MRI scanner, as we wanted our paradigm to have high ecological validity by mimicking situations encountered in everyday life, when we may overhear a conversation and are not asked to explicitly pay attention or remember what was said. By explicitly asking participants to carefully listen and try to remember the conversations, any differences in overall discriminative accuracy between diagnostic groups would have likely been further reduced. Indeed, previous studies where direct attentional cues were provided to ASD youth have shown increased brain activity and improved behavioral performance as compared to conditions where such instructions were not given (24, 41). Importantly, both neurotypical youth and youth with ASD had higher discriminative accuracy for conversations than noises when these were each presented alone, as well as higher discriminative accuracy when identifying conversations than noises when conversations and noises were presented simultaneously. In addition, both neurotypical and ASD youth showed the expected pattern whereby accuracy in identifying topics of conversation was poorer for conversations presented over background noise than for conversations presented alone. Although this latter difference was not statistically significant when using d' collapsed across the easy (yes/no) and hard (multiple-choice) questions, when looking at percent accuracy for the harder multiple-choice questions, ASD youth performed significantly worse in the conversation-in-noise condition than in the conversation alone condition, a pattern not observed in TD youth. Overall, these findings are in agreement with previous work in adults and adolescents with ASD showing that recall is poorer for sentences presented simultaneously with background sounds (22, 23). However, our findings of similar discriminative accuracy (d') between typically-developing and ASD youth when identifying conversations heard in the context of background noises are in contrast to previous work suggesting that individuals with ASD are poorer at discriminating speech-in-noise relative to their neurotypical counterparts (22, 23). This difference may in part be explained by our choice of noise stimuli, which were deliberately chosen to be only mildly aversive and, unlike those used in prior studies, also easily recognizable. Indeed, this methodological choice may also explain why we did not observe between-group differences in brain regions previously implicated in processing aversive auditory stimuli (e.g., amygdala, thalamus, auditory cortex), which have previously been documented in ASD participants (24, 31, 32, 42). Importantly, the lack of significant between-group differences in brain responses to mildly aversive noises in this study may also in part reflect the more stringent statistical threshold employed in the current study, in keeping with evolving standards in the neuroimaging field (43). Indeed, at more liberal thresholds we too observed greater activity in the amygdala and primary auditory cortex during exposure to mildly aversive noise in ASD youth as compared to TD youth.

At the neural level, typically-developing and ASD youth showed overall similar patterns of brain activity when listening to conversations alone, noises alone, and conversations shrouded in noise. The only significant between-group difference was detected when comparing brain activity observed when youth were presented with conversations and environmental noises simultaneously *versus* conversations alone. Here, the addition of background noise to conversations elicited greater activity in the precuneus and primary visual cortex in ASD relative to TD youth. The precuneus is a canonical hub of the default mode network, a network of brain regions implicated in thinking about the self and others (44) and narrative comprehension in neurotypical adults (45, 46). Our finding of increased activity in visual cortex during auditory stimulation in ASD youth, relative to typically-developing youth, is consistent with previous findings in individuals with ASD showing increased brain activity in the visual system during semantic decision making (47) as well as auditory pitch discrimination (48), suggesting atypical integration of auditory and visual sensory systems in ASD (42, 49). Our findings thus suggest that similar behavioral profiles may in part reflect processing differences at the neural level whereby the challenging task of listening to social interactions over background noise requires activation of additional brain regions in youth with ASD, relative to neurotypical controls.

The ability to deploy attention to socially meaningful information rests on being able to divert attention away from less relevant distracting stimuli; accordingly, in an attempt to hone in on the neural substrates of social attention, we next sought to identify brain activity that was related to the successful encoding of the topics of conversation. More specifically, we examined how brain responses while participants listened to conversations in the context of background noise (above and beyond brain responses associated with attending to conversations and noises alone) predicted later recognition of what was heard. In both neurotypical youth and youth with ASD, greater accuracy in identifying the topics of conversations heard in the context of background noise was predicted by greater activity in left hemisphere voice-selective cortex. Previous work in neurotypical adults has shown that this voice-selective region preferentially responds to vocal stimuli, and that activity in this region decreases when voice stimuli are masked by background noise (19, 20). Thus, heightened activity in this region when listening to conversations shrouded in common environmental noises may serve as a compensatory mechanism, allowing both youth with and without ASD to focus their attention on the socially-relevant information in the presence of distracting auditory stimuli. Importantly, better recognition accuracy in youth with ASD was also associated with greater activity in a wider network of brain regions implicated in language processing. Indeed, relative to typically-developing youth, ASD youth showed significantly greater activity in left-hemisphere angular gyrus. This region plays an important role in language comprehension (50–52) and prior work shows that disrupting activity in this area reduces the ability to comprehend speech under difficult listening conditions (53). The angular gyrus is also an important region for theory of mind (TOM)—the ability to understand the actions and thoughts of others (54, 55). TOM is a critical skill in reasoning about others' state of mind and plays a role in high-level language processing including the use and understanding of language within a social environment (56). Thus, similar to the heightened response in the voice-selective-region observed in both neurotypical and ASD youth, this increased activity in speech processing cortex in youth with ASD could reflect compensatory processes resulting in improved sensitivity to speech stimuli, thereby boosting youths' ability to encode and later accurately discriminate between conversation topics heard over background noise. If this interpretation is correct, individual differences in responsivity observed in this region in the context of our paradigm should be associated with the more general ability to hone in on socially-relevant information, and ultimately result in less severe social impairments. Consistent with this hypothesis, neural activity in this speech-processing region while participants listened to conversations shrouded in noise was associated with better social motivation and social cognition in youth with ASD.

This study has several limitations. First, due to the correlational nature inherent to all neuroimaging studies, while we hypothesized that the increased activity in language-related and TOM regions allowed ASD youth to hone in on socially relevant information, we cannot rule out the alternative account that greater activity in these brain regions merely resulted from more successful processing of language through noise. Second, atypical heightened sensitivity to sensory stimuli (known as sensory over-responsivity; SOR) affects over half of children with ASD (26, 27) and is an important contributor to altered processing of both social and non-social stimuli in youth with ASD (24, 31, 32, 42); however, given our small sample size, we were unable to directly compare groups of ASD youth with and without SOR. More work is needed to understand how SOR may mediate neural responses to ecologically valid social and non-social stimuli in the environment. Importantly, recent work also suggests that there may be sex-differences in the development of multisensory speech processing in TD and ASD youth (57); thus, examining the interaction between sex, sensory processing, and social cognition is an important direction for future research. In addition, participants in our study were all high-functioning individuals who developed language and had verbal IQ in the normal range, making it more likely that our participants would have the ability to hone in on social stimuli compared to more affected individuals. In future studies it will be crucial replicate these findings and to extend this work to individuals with more severe ASD phenotypes, as well as to younger children on the autism spectrum. To this end, prospective studies of infants at high risk for developing ASD will be essential to track the longitudinal co-development of sensory responsivity, language acquisition, and ASD symptomatology.

To conclude, using a novel and ecologically valid paradigm, here we sought to better understand the neural correlates of social attention. Our findings indicate youth with ASD who successfully encoded socially-relevant information in the presence of distracting stimuli did so by up-regulating activity in neural systems supporting speech and language processing, thus suggesting that focusing on both social and non-social stimuli simultaneously may be more of a challenge for ASD youth relative to their neurotypical counterparts. This work buttresses the importance of further examining the relationship between social attention and sensory processing atypicalities, particularly early in development, to shed new light on the onset of autism symptomatology, as well as to inform the design of novel interventions.

## Data Availability Statement

The datasets generated for this study are available on request to the corresponding author.

## Ethics Statement

The studies involving human participants were reviewed and approved by University of California, Los Angeles Institutional Review Board. Written informed consent to participate in this study was provided by the participants' legal guardian/next of kin.

## Author Contributions

This study was conceived of and designed by LH, SG, KL, JL, SB, and MD. Data acquisition was performed by LH, KL, and JL. Data analysis was completed by LH and MI. All authors contributed to data interpretation and drafting of the manuscript, and provided critical feedback on the manuscript and its intellectual content.

## Funding

This work was supported by the National Institute of Mental Health (grants R01MH100028 and K08 MH112871) and the Simons Foundation Autism Research Initiative (grant 345389). Some of the authors were supported by training grants/fellowships from the National Institute of Neurological Disorders and Stroke (F99 NS105206 to LH, T32 NS048004 to LH and KL), the National Institute of Mental Health (F32 MH105167 to SG, F31 MH110140 to KL), the National Institute of Child Health and Human Development (F31 HD088102 to JL), and the Roche/ARCS Foundation Scholar Award Program in the Life Sciences (KL and JL). The project was also in part supported by grants RR12169, RR13642, and RR00865 from the NIH National Center for Research Resources. We are also grateful for generous support from the Brain Mapping Medical Research Organization, Brain Mapping Support Foundation, Pierson-Lovelace Foundation, The Ahmanson Foundation, the William M. and Linda R. Dietel Philanthropic Fund at the Northern Piedmont Community Foundation, the Tamkin Foundation, the Jennifer Jones-Simon Foundation, the Capital Group Companies Charitable Foundation, the Robson Family, and the Northstar Fund. The contents of this paper are solely the responsibility of the authors and do not necessarily represent the official views of the National Institutes of Health.

## Conflict of Interest

The authors declare that the research was conducted in the absence of any commercial or financial relationships that could be construed as a potential conflict of interest.
